# Two complete genome sequences of antimonite-oxidizing bacteria, *Mesorhizobium* sp. strain ANAO-SY3R2 and *Hydrogenophaga* sp. strain ANAO-22, isolated from mine tailing soil

**DOI:** 10.1128/mra.01073-24

**Published:** 2024-12-31

**Authors:** Yoriko Yamashita, Natsuko Hamamura

**Affiliations:** 1 Graduate School of Systems Life Sciences, Kyushu University, Fukuoka, Japan; 2 Department of Biology, Faculty of Science, Kyushu University, Fukuoka, Japan; SUNY College of Environmental Science and Forestry, Syracuse, New York, USA

**Keywords:** antimony oxidation, *Mesorhizobium*, *Hydrogenophaga*

## Abstract

We report the complete genome sequences of two antimony-oxidizing bacteria, *Mesorhizobium* sp. strain ANAO-SY3R2, comprising one chromosome (4.3 Mbp) and four circular plasmids, and *Hydrogenophaga* sp. strain ANAO-22, comprising one chromosome (5.8 Mbp) and two circular plasmids. These genome information extend our understanding of physiological versatility of antimony-transforming microorganisms.

## ANNOUNCEMENT

Antimony (Sb) is a naturally occurring toxic metalloid. Although the concentrations of Sb in soils are generally low, elevated levels of Sb have been introduced through mining and other anthropogenic activities (1). Despite its toxicity, microorganisms have evolved mechanisms for resistance and redox transformations of Sb (2–5).

Antimonite-oxidizing bacteria were isolated from Sb-contaminated wet sediment collected from a depth range of 6–9 cm at former mine site (Ehime, Japan; 33°53'20.4"N, 133°12'51.6"E). Details of the sampling site have been described previously (6). Enrichment cultures were established under microaerobic conditions by inoculating ~1 g of the sediment into basal salts medium (50 mL medium in 160 mL serum bottles) (4) amended with 1 mM Sb(III) [as potassium antimony(III) tartrate], 5 mM HCO^3-^, and 0.002% (wt/vol) yeast extract. The bottle headspace was replaced with N_2_ gas, and filter-sterilized oxygen was added to achieve a final concentration of 1% (vol/vol). The bottles were sealed with butyl rubber stoppers and aluminum crimp caps and incubated at 25°C in the dark without shaking. After several transfers, pure cultures were obtained by repeated dilution of positive enrichments in the agar shake medium prepared by the addition of purified agar (1.0% [wt/vol]) to the basal salts medium described above. The obtained isolates were identified as *Mesorhizobium* sp. and *Hydrogenophaga* sp. via 16S rRNA gene sequencing as described previously (7) and designated strain ANAO-SY3R2 and strain ANAO-22, respectively.

Both strains were grown in the basal salts medium with Sb(III) under microaerobic conditions as previously described. DNA was extracted by using Blood and Cell Cultures DNA Midi Kit (Qiagen) and purified with the DNeasy PowerClean Pro Cleanup Kit (Qiagen) following the manufacturer's protocols. The extracted DNA was sheared into 10- to 20-kb fragments using g-TUBE devise (Covaris, Massachusetts, USA), and size selection (cutoff, 10 kb) was performed using the BluePippin system (Saga Scientific, Massachusetts, USA). Libraries were prepared using a single-molecule real time (SMRT) cell 8Pac V3 and the P6 DNA polymerase binding kit (PacBio, California, USA), following the manufacturer's instruction. Genome sequencing was performed on PacBio RSII sequencing platform (PacBio, California, USA) at Macrogen Japan (Macrogen Corp., Japan). High-fidelity (Hi-Fi) reads with quality values of >20 or 99% accuracy were acquired by the SMRT Portal (v2.3). The filtered raw reads were assembled to generate contigs without any gaps, and contigs were connected to form a circular contig when the end of the contigs was overlapped using the Hierarchical Genome Assemble Process (HGAP) v3.0 with default parameters. Subread counts were 105,644 (mean length, 10,021 bp; *N_50_,* 14,835 bp) and 185,681 (mean length, 9,223 bp; *N_50_,* 14,287 bp) comprising a total of 1,058,681,081 and 1,712,692,047 bp for *Mesorhizobium* and *Hydrogenophaga* isolates, respectively.

The assembled genome of *Mesorhizobium* sp. ANAO-SY3R2 consists one circular chromosome (4,338,903 bp with 63.2% GC contents) and four circular plasmids (410,557 bp, 355,698 bp, 189,652 bp, and 72,341 bp, with GC contents of 63.8%, 61.6%, 61.2%, and 64.2% respectively). Average nucleotide identity (ANI) was calculated using OrthoANI (8), and the genome of strain ANAO-SY3R2 showed ANI values of 77.58% and 82.88% with phylogenetically closely related *Mesorhizobium australicum* WSM2073 (16S rRNA gene identity, 98.7%) and *Aminobacter aminovorans* DSM7048 (16S rRNA gene identity, 99.1%) genomes, respectively. The genome of *Hydrogenophaga* sp. ANAO-22 comprises one circular chromosome (5,848,526 bp with 67.7% GC contents) and two circular plasmids (86,795 bp and 72,339 bp, with GC contents of 65.6% and 64.2%, respectively). The ANI values for the strain ANAO-22 were 85.77% and 79.68% with closely related *Hydrogenophaga palleronii* NBRC 102513 (16S rRNA gene identity, 99.4%) and *H. flava* NBRC 102514 (16S rRNA gene identity, 97.8%) genomes, respectively.

Genome annotation was conducted using the Prokaryotic Genome Annotation Pipeline v 6.7 (9), yielding 4,970 and 5,681 protein-coding DNA sequences, 57 and 43 tRNAs, and nine (three copies of 16S, 23S, and 5S) and three (one copy of 16S, 23S, and 5S) rRNAs for *Mesorhizobium* and *Hydrogenophaga* isolates, respectively.

Further details on Sb(III) oxidation by these newly isolated strains will be reported in future studies.

## Data Availability

The complete genome sequence of *Hydrogenophaga* sp. str. ANAO-22 was deposited in GenBank under accession numbers CP159956, CP159957, and CP159958; BioProject accession number PRJNA622630; BioSample accession number SAMN41832008; and SRA accession number SRR29429604. *Mesorhizobium* sp. str. ANAO-SY3R2 was deposited under accession numbers CP159951, CP159952, CP159953, CP159954, and CP159955; BioProject accession number PRJNA622630; BioSample accession number SAMN41831677; and SRA accession number SRR29430160.
